# Fatal acute-on-chronic liver failure following camrelizumab for hepatocellular carcinoma with HBsAg seroclearance: a case report and literature review

**DOI:** 10.3389/fmed.2023.1231597

**Published:** 2023-08-14

**Authors:** Fenghui Li, Tao Wang, Fei Tang, Jing Liang

**Affiliations:** Department of Gastroenterology and Hepatology, The Third Central Hospital of Tianjin, Tianjin Key Laboratory of Extra-corporeal Life Support for Critical Diseases, Artificial Cell Engineering Technology Research Center, Tianjin Institute of Hepatobiliary Disease, Tianjin, China

**Keywords:** immune checkpoint inhibitors (ICIs), immune-mediated liver injury caused by checkpoint inhibitors (ILICI), hepatitis B, clearance of HBsAg, liver failure

## Abstract

In the last few years, immune checkpoint inhibitors (ICIs) have become major therapeutic agents for the treatment of advanced hepatocellular carcinoma (HCC). However, immunotherapy can activate hepatitis B virus (HBV), and immune clearance may lead to liver failure and even life-threatening conditions. Here we report a case of HCC with HBV-related cirrhosis that caused severe liver injury and rapidly progressed to fatal acute-on-chronic liver failure (ACLF) after only once application of camrelizumab; the patient underwent serological conversion of hepatitis B surface antigen (HBsAg) with liver injury. The patient’s condition progressed rapidly. We added corticosteroids and applied plasma dialysis, along with tenofovir alafenamide (TAF) to control HBV. However, the patient eventually died of liver failure. To our knowledge, there are few reports of HBsAg clearance due to ICIs accompanied by fatal acute-on-chronic liver failure shortly after ICIs initiation. These results suggest that ICIs can cause fatal liver injury in a short term; in patients with chronic HBV infection, ICIs use may promote serological conversion of HBsAg.

## 1. Background

Immune checkpoint inhibitors (ICIs) have dramatically improved the survival rate of patients with advanced tumors. However, with their increasing use, the incidence of immune-related adverse events (irAEs) has also increased, possibly involving any organs, including the liver (1). Immune-mediated liver injury caused by checkpoint inhibitors (ILICI) ranges from mild to liver failure, which is life-threatening. ILICI is the third type of drug-induced liver injury caused by the indirect effect of ICIs on the liver through their immune-mediated mechanism of action (2).

ICIs may theoretically exhibit both positive and negative effects on hepatitis B virus (HBV)–related pathophysiology. Some studies have suggested that HBV reactivation occurs in patients undergoing anti-programmed cell death protein-1 (PD-1) therapy (3, 4). In the CheckMate 040 trial, 3 (6%) of 51 HBV-related hepatocellular carcinoma (HCC) patients experienced a 1-log reduction in hepatitis B surface antigen (HBsAg) levels during nivolumab treatment (5). Therefore, ICIs may promote HBsAg clearance or HBV reactivation. Few cases of liver failure caused by HBsAg clearance by ICIs have been reported (6, 7).

Here we report the case of a patient with HBV-related HCC who was treated one time with camrelizumab and developed grade 4 hepatitis that progressed to fatal acute-on-chronic liver failure (ACLF) accompanied by HBsAg seroclearance.

## 2. Case presentation

A 47-year-old man with a history of HBV-related cirrhosis received entecavir antiviral therapy for 1 year and discontinued it for 2 years. He discontinued entecavir due to the unavailability of drugs during the prevalence of novel coronary pneumonia and poor adherence. He had no known history of other diseases such as hypertension, diabetes, habitual alcohol use, or blood transfusion. On December 14, 2022, he visited a local hospital reporting discomfort in the right upper abdomen, and enhanced magnetic resonance imaging examination indicated a tumor in the right hepatic lobe accompanied by lymph node metastases in the retroperitoneum. At the same time, tenofovir alafenamide (TAF) was given.

**Figure 1:**
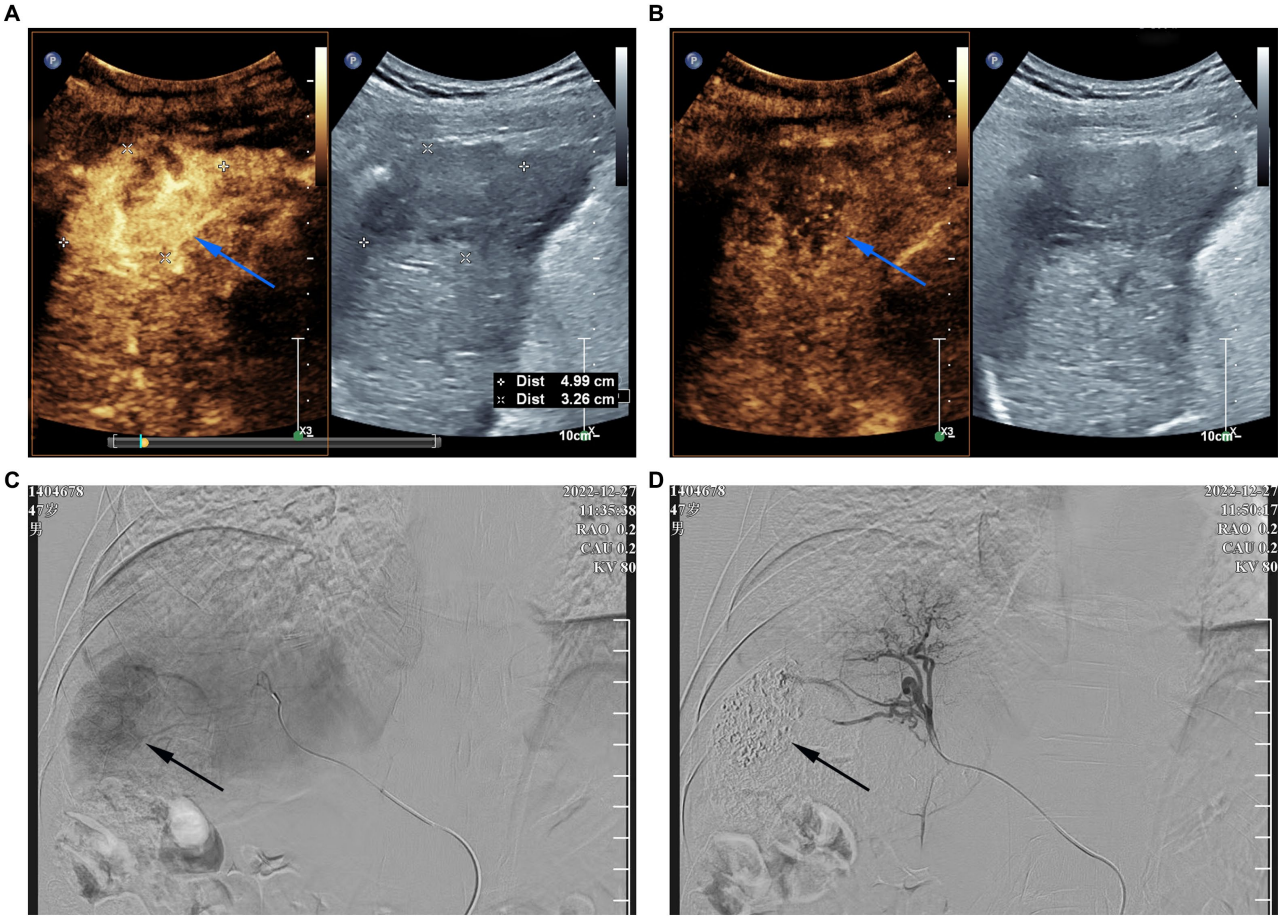
Contrast-enhanced ultrasound (CEUS) images and digital subtraction arteriogram (DSA) during TACE **(A)** CEUS in the arterial phase shows hyperenhancement of the mass [**(A)** arrow]; **(B)** The mass shows washout in the portal venous phase of CEUS [**(B)** arrow]; **(C)** pre-embolization DSA of the mass, black arrow represents tumor blush; **(D)** post-embolization DSA was repeated and demonstrated no residual enhancing tumor.

The patient was transferred to our hospital on December 26, 2022. Routine blood and liver function test results were shown in Table 1. Assay testing for HBV infection was positive for HBsAg, hepatitis B e antigen (HBeAg), and hepatitis B core antibody (HBcAb)and negative for hepatitis B surface antibody (HBsAb), hepatitis B e antibody (HBeAb), and HBV DNA (4.19 × 10^4^ IU/mL). Liver tests were negative for other hepatitis viruses (such as hepatitis C virus [HCV]), autoimmune liver diseases, and metabolic liver diseases such as hepatolenticular degeneration and hemochromatosis. The patient’s alpha-fetoprotein (AFP) level was normal. Contrast-enhanced ultrasound (CEUS) was also performed (Figures 1A,B). According to the AASLD treatment guidelines (8), the patient was diagnosed as hepatocellular carcinoma with lymph node metastases in the retroperitoneum. He was staged at Barcelona Clinic Liver Cancer C, Child-Pugh grade A, and an Eastern Cooperative Oncology Group Performance Score 0. Our patient with liver-dominant disease and with limited lymph node metastases, transarterial chemoembolization (TACE) was performed according to the ISMIO treatment guidelines (9) (Figures 1C,D). After TACE, the patient’s liver function was not impaired and camrelizumab was administered (200 mg intravenous drip).

**Table 1:** Clinical indicators.

Variables	First hospitalization (December 26, 2022)	Second hospitalization (January 18, 2023)	Third hospitalization (January 26, 2023)	Fourth hospitalization (February 10, 2023)
PT (s)	17.00	19.10	20.90	39.70
INR	1.38	1.61	1.84	4.21
WBC (×10^9^/L)	4.76	2.57	3.87	31.93
NEUT (%)	70.5	62.2	69.7	89.3
HGB (g/L)	124	114	121	90
PLT (×10^9^/L)	107	55	42	45
ALB (g/L)	35.9	27.2	28.8	25.7
ALT (U/L)	14	127	300	143
AST (U/L)	28	1,483	722	249
ALP (U/L)	178	217	216	149
GGT (U/L)	131	285	155	63
TBil (mg/dL)	1.68	3.36	21.84	34.25
DBIL(mg/dL)	0.18	2.13	16.88	27.68
Cr (mg/dL)	0.80	0.71	1.05	3.54

PT, prothrombin time; INR, international normalized ratio; WBC, white blood cell; NEUT, neutrophil; HGB, hemoglobin; PLT, platelet; ALB, albumin; ALT, alanine aminotransferase; AST, aspartate aminotransferase; ALP, alkaline phosphatase; GGT, gamma-glutamyltransferase; TBIL, total bilirubin; DBIL, direct bilirubin; Cr, creatinine.

Three weeks later (January 18, 2023), the patient underwent liver function tests and then re-administered camrelizumab immediately. His liver function tests (Table 1) suggested the presence of grade 4 ILICI. The Roussel Uclaf Causality Assessment Method (RUCAM) was calculated to be +7.

**Figure 2:**
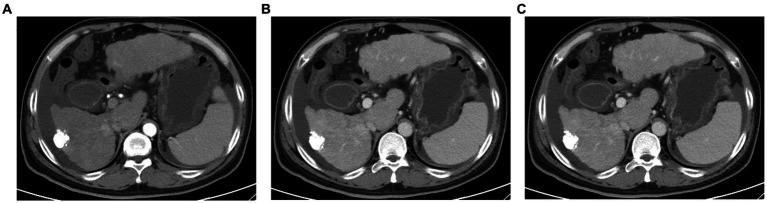
Abdominal enhanced computed tomography images **(A)** Plain CT scan; **(B)** arterial phase of contrast-enhanced CT; **(C)** portal vein phase of contrast-enhanced CT. CT examination of the abdomen show no biliary obstruction and disease progression of intrahepatic lesions.

On January 26, 2023, the patient was readmitted to our hospital with a 1-week history of yellowing of the skin and sclera. He also experienced severe weakness, loss of appetite, abdominal distention, and pain in the left lumbar region. A physical examination revealed severe jaundice, positive liver palm and spider nevus; no obvious abnormal findings during cardiopulmonary auscultation; a soft abdomen; pain at the left upper ureteral pressure point; percussion pain in the renal area; no significant enlargement of the liver or spleen on palpation; negative mobility dullness; and no edema of either leg. Routine blood and liver function test results were shown in Table 1. Serological tests for hepatitis A virus, hepatitis E virus, human immunodeficiency virus, cytomegalovirus, Epstein–Barr virus were negative. The patient’s autoantibodies were normal. HBV testing was negative for HBsAg but positive for HBsAb. Simultaneously, we analyzed lymphocyte subgroups in the peripheral blood using flow cytometry and found that the proportions of helper T cells and cytotoxic T cells were significantly reduced, whereas that of B lymphocytes was increased. Contrast-enhanced computed tomography of the abdomen, which excluded biliary obstruction and disease progression of the intrahepatic lesions (Figure 2), suggested the presence of a stone in the upper left ureter with dilatation of the upper ureter. This may have been related to the patient’s left lumbar pain. Because the patient’s liver function tests suggested hepatocellular damage and stasis of bile, carrilizum was stopped immediately, intravenous magnesium isoglycyrrhizinate 200 mg once a day, oral ursodeoxycholic acid (UDCA) 250 mg three times a day were given. In addition, he had a ureteral stone and urinary tract infection, so we gave him intravenous ertapenem 1 g once a day to fight the infection and performed transurethral stenting to relieve the urethral obstruction.

**Figure 3:**
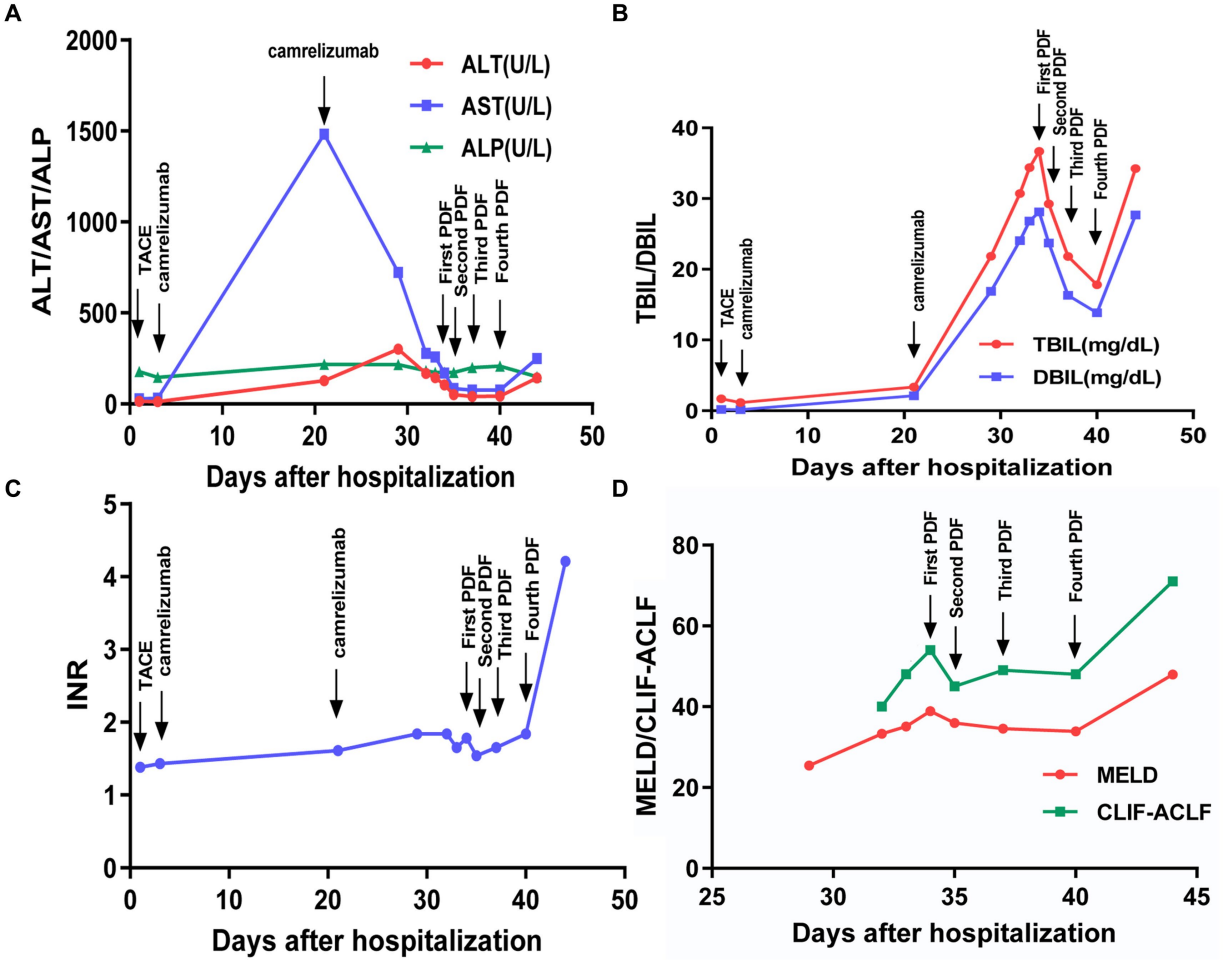
Changes of various indicators after hospitalization. **(A–C)** Trend of clinical indicators; **(D)** trend of severity scores.

Over the next 5 days, the patient’s condition deteriorated rapidly and progressed to ACLF, with new-onset of encephalopathy, acute kidney injury, and acidosis. Methylprednisolone (80 mg/day) was added and plasma dialysis (PDF) was performed four times. The patient’s liver function improved and he felt relief from the abdominal distension and weakness (Figures 3A–C). However, the patient refused to remain hospitalized.

On February 10, 2023, the patient was admitted to the intensive care unit with severe infection, ACLF, renal failure, and lactic acidosis (Table 1). At last, the patient’s Chronic Liver Failure Consortium (CLIF-C) ACLF score was calculated to be 71, and the model for the end-stage liver disease (MELD) score was 48, indicating an unfavorable outcome (Figure 3D). Ascitic fluid tests suggested ascites infection. Despite aggressive treatment, the patient’s condition continued to deteriorate and he eventually died. The causes of death included hepatic failure, renal failure, and infectious shock.

## 3. Discussion

As ICIs are widely used to treat solid tumors, an increasing number of irAEs have been reported. The incidence of ILICI is 3–15%, while that of grade 3 and 4 ILICI is 1.7 and 3%, respectively (10, 11). Acute liver failure is rare (6). Few studies to date examined fatal hepatitis caused by ICIs, most cases of which involved acute liver failure without a history of chronic liver disease (12, 13). When ICIs were administered, the incidence of HBsAg clearance or HBV reactivation accompanied by liver failure was extremely low (6, 7).

Approximately 80% of HCC patients are in intermediate or advanced stages at diagnosis, leading to poor prognosis (14). TACE and systemic therapy are standard treatments for patients with intermediate and advanced HCC, respectively (15). For patients with limited extrahepatic metastasis and with liver-dominant disease, TACE may play a role in controlling the intrahepatic tumors (16). ICIs have shown dramatic effects to the treatment of advanced HCC patients (17). Camrelizumab is used for various malignancies including HCC, which is the first-line treatment for advanced HCC in China (18). Combined therapy with different mechanisms may improve outcomes. Here we reported the case of a patient with HCC who had a history of HBV-related cirrhosis and treated with TACE combined with Carrilizum. Three weeks after the initial camrelizumab infusion, the patient experienced serious adverse hepatic event with HBsAg seroclearance that eventually led to his death.

Immune checkpoints that have received considerable attention include cytotoxic T-lymphocyte-associated protein-4 (CTLA-4), PD-1, and programmed cell death ligand 1 (PD-L1) (19). Each promotes immune tolerance and helps tumor cells escape immune responses. When ICIs block these proteins, their inhibitory effect is released, leading to T cell activation and proliferation, ultimately killing the tumor cells (20). HBV-specific T cells in patients with chronic HBV infection are difficult to detect and exhibit a dysfunctional state called “failure” (21). This leads to impaired cytokine production and the sustained expression of multiple inhibitory receptors such as PD-1 and CTLA-4 (22). The inhibition of PD-1/PD-L1 signaling may enhance antiviral-specific T cell responses, which is the key to achieving HBsAg clearance (23). Therefore, in chronic HBV infection, blocking receptors for PD-1, PD-L1, or CTLA-4 using ICIs may play a role in restoring the function of these depleted T cells (24). Therefore, ICIs treatment can disrupt the immune homeostasis in chronic HBV infection, leading to HBV reactivation or HBsAg clearance. For this patient, immune clearance may be the cause of liver damage. In addition, his RUCAM was calculated to be +7, indicating carrilizum to be a probable cause of liver injury.

In virally suppressed HBeAg-negative patients, checkpoint blockade leads to a decline in HBsAg (7). A few patients reportedly experienced a significant elevation in alanine aminotransferase levels accompanied by HBsAg seroconversion that were considered immune-mediated HBV flares rather than ILICI (7). ICIs may be associated with HBsAg seroclearance; however, they may also be potentiated with HBV reactivation. Wong et al. reported that HBV reactivation occurred in only two of 990 patients treated with ICIs, while HBsAg seroreversion was observed in <1% of the study population (25). Yoo et al. reported that, in 3465 patients treated with ICIs against tumors, the incidence of HBV reactivation was 0.14%, while HBsAg seroreversion occurred in 0.4% of HBsAg-positive patients (3). When a PD-1 inhibitor was administered, HBsAg clearance occurred in our patient along with fatal ACLF. We reviewed case studies and retrospective analysis of hepatitis B patients with ICIs, some of which are shown in Supplementary Table S1.

Furthermore, the B cell response may also play an important role in controlling HBV infection. HBsAg-specific B cells were detected in HBV-vaccinated individuals but not in patients with chronic HBV infection (26). Other studies suggested that HBsAg-specific B cells from patients with chronic hepatitis B are functionally defective B cells with high inhibitory receptor PD-1 expression and are unable to secrete HBsAb. PD-1 blockage contributes to the recovery of B cell function (27). Our patient had an increased proportion of B lymphocytes, possibly related to the presence of HBsAb.

Studies have suggested that HBV reactivation rarely occurs in patients receiving prophylactic antiviral therapy during ICIs treatment. However, HBV reactivation may occur in HBsAg-positive patients who do not receive or not regular receive antiviral therapy (3). Therefore, screening for HBV before and during ICIs treatment and the preventive use of antiviral drugs in high-risk patients with HBV reactivation are crucial. Unfortunately, our patient with HBV-related HCC who was treated with camrelizumab accompanied by HBsAg seroclearance and developed to fatal acute-on-chronic liver failure (ACLF).

For patients with chronic HBV infection, if severe hepatic injury occurs after ICIs application, corticosteroids can be considered in addition to timely ICIs discontinuation. Pandey et al. reported the case of a lung cancer patient with positive HBsAg in whom HBV reactivation occurred after use of pembrolizumab, resulting in severe liver damage. After treatment with steroids and antiviral drugs for HBV infection, the patient’s liver function gradually returned to normal (28). However, whether corticosteroids increase the risk of HBV reactivation and improve the survival of patients with fulminant liver failure remain unknown. Our patient had HBV-related cirrhosis and positive HBV-DNA due to lack of regular antiviral treatment. Simultaneously, he had infection for which systemic application of corticosteroids may led to HBV reactivation and infection exacerbation (29). HBV reactivation can lead to fatal liver injury; therefore, we did not immediately administer corticosteroids when the patient developed to ILICI. However, his condition worsened rapidly and ACLF occurred. Thus, we added methylprednisolone on the premise of administering antibiotics for infection and TAF to control HBV. Our patient had rapidly progressing liver failure with hepatic encephalopathy, renal failure, and infection; therefore, we treated this patient with PDF and methylprednisolone, and his liver function improved. However, due to rapid disease progression and treatment interruption, the patient eventually died of multiple organ failure.

## 4. Conclusion

In conclusion, the probability of HBsAg seroreversion is extremely low under natural HBV infection status and existing treatment regimens (30). ICIs disrupt immune homeostasis and improve immune depletion in patients with chronic HBV infection, which may contribute to HBsAg seroreversion. In patients with HBV-related HCC, ICIs use may promote HBsAg clearance, leading to fatal ACLF. Therefore, aggressive antiviral therapy and close monitoring are important for patients with HBV-related HCC.

## Data availability statement

The original contributions presented in the study are included in the article/Supplementary material, further inquiries can be directed to the corresponding author.

## Ethics statement

Written informed consent was obtained from the individual(s) for the publication of any potentially identifiable images or data included in this article.

## Author contributions

FL and TW writing – original draft preparation and both contributed equally to the study. FT data collection and analysis. JL writing – reviewing and editing. All authors contributed to the article and approved the submitted version.

## Funding

This study was supported by Tianjin Key Medical Discipline (Specialty) Construction Project (TJYXZDXK-034A).

## Conflict of interest

The authors declare that the research was conducted in the absence of any commercial or financial relationships that could be construed as a potential conflict of interest.

## Publisher’s note

All claims expressed in this article are solely those of the authors and do not necessarily represent those of their affiliated organizations, or those of the publisher, the editors and the reviewers. Any product that may be evaluated in this article, or claim that may be made by its manufacturer, is not guaranteed or endorsed by the publisher.
