# Supplementary material for: Fatal acute-on-chronic liver failure following camrelizumab for hepatocellular carcinoma with HBsAg seroclearance: a case report and literature review
Source: Front Med (Lausanne). 2023 Aug 14;10:1231597. doi: 10.3389/fmed.2023.1231597 (PMC10461443; doi:10.3389/fmed.2023.1231597)
Supplement: Supplementary file 1 [file Data_Sheet_1.PDF]

Table S1. Review of previous reports

	Author	Year	Journal	Title	Doi
1	Hong	2023	Journal of Clinical Oncology	Immune checkpoint inhibitors use and the incidence of hepatitis B virus reactivation or immune-related hepatitis in non-small cell lung cancer patients with chronic hepatitis B	10.1200/JCO.2023.41.16_suppl.9086
2	Nardo	2023	Oncologist	Safety and Efficacy of Immune Checkpoint Inhibitors in Patients with Cancer and Viral Hepatitis: The MD Anderson Cancer Center Experience	10.1093/oncolo/oyad039
3	Lei	2023	Hepatol Int	Comparison of hepatitis B virus reactivation in hepatocellular carcinoma patients who received tyrosine kinase inhibitor alone or together with programmed cell death protein-1 inhibitors	10.1007/s12072-022-10450-4
4	Hagiwara	2022	Hepatol Res	Clinical implication of immune checkpoint inhibitor on the chronic hepatitis B virus infection	10.1111/hepr.13798
5	Lin	2022	Cancer Immunol Immunother	Hepatotoxicity associated with PD-1 blockade antibodies in cancer patients co-infected with hepatitis B virus	10.1007/s00262-021-03082-4
6	Wong	2021	Am J Gastroentero	Hepatitis Flare During Immunotherapy in Patients With Current or Past Hepatitis B Virus Infection	10.14309/ajg.0000000000001142
7	Xu	2021	Transl Lung Cancer Res	Safety and efficacy of anti-PD-1 inhibitors in Chinese patients with advanced lung cancer and hepatitis B virus infection: a retrospective	10.21037/tlcr-21-79

				single-center study	
8	Lee	2020	J Immunother Cancer	Risk of HBV reactivation in patients with immune checkpoint inhibitor-treated unresectable hepatocellular carcinoma	10.1136/jitc-2020-001072
9	Chan	2020	Lung Cancer	Immune checkpoint inhibition for non-small cell lung cancer in patients with pulmonary tuberculosis or Hepatitis B: Experience from a single Asian centre	10.1016/j.lungcan.2020.05.020
10	Kundumadam	2020	Cureus	Pembrolizumab-Induced Immune-Mediated Hepatitis and Concurrent Hepatitis B Reactivation in a Patient With Non-Small Cell Lung Cancer	10.7759/cureus.11522
11	Zhang	2019	J Immunother Cancer	Hepatitis B virus reactivation in cancer patients with positive Hepatitis B surface antigen undergoing PD-1 inhibition	10.1186/s40425-019-0808-5
12	Pandey	2018	Case Rep Oncol Med	A Rare Case of Pembrolizumab-Induced Reactivation of Hepatitis B	10.1155/2018/5985131
13	Kothapalli	2018	Melanoma Res	Safety and efficacy of anti-PD-1 therapy for metastatic melanoma and non-small-cell lung cancer in patients with viral hepatitis: a case series	10.1097/CMR.0000000000000434
14	Koksal	2017	Ann Oncol	HBV-related acute hepatitis due to immune checkpoint inhibitors in a patient with malignant melanoma	10.1097/CMR.0000000000000434
15	Ragunathan	2017	Am J Gastro	Hepatitis B Reactivation After Administration of Pembrolizumab (KEYTRUDA): a unique case report	

16	Talotta	2016	BMC Pharmacol Toxicol	Reactivation of occult hepatitis B virus infection under treatment with abatacept: a case report	10.1186/s40360-016-0060-2
